# Strontium-89 therapy for the treatment of huge osseous metastases in prostate carcinoma: A case report

**DOI:** 10.3892/etm.2012.807

**Published:** 2012-11-12

**Authors:** WENJIE ZHANG, WEIWEI ZHAO, ZHIYUN JIA, HOUFU DENG

**Affiliations:** Department of Nuclear Medicine, West China Hospital of Sichuan University, Chengdu, Sichuan 610041, P.R. China

**Keywords:** strontium-89, radionuclide, huge osseous metastases, nuclear medicine

## Abstract

Prostate cancer is a growing public health problem. The palliation of pain in patients with painful bone metastases is of primary importance in the clinical management of advanced cancer. Internal therapy with radionuclides, which concentrate at sites of increased bone turnover, is used to control pain and improve quality of life as an alternative to conventional therapies. In the present study, we report the case of a 52-year-old male who had been diagnosed with prostate cancer. The patient presented with severe pain in multiple areas, but particularly in the right hip. A whole-body bone scan revealed that the right hip, ilium and ischium were covered with huge metastatic lesions. Treatment with radionuclide strontium-89 chloride (^89^Sr) resulted in a partial response which was confirmed by the successful relief of pain and other imaging modalities. No significant change in the leukocyte or thrombocyte levels was observed. The results of the present study indicate that systemic radionuclide therapy using ^89^Sr is an effective, well-tolerated and safe palliative treatment in patients with huge osseous metastases in prostate carcinoma.

## Introduction

Prostate cancer is the most common malignancy and the second leading cause of cancer-associated mortality in males (1). Bone metastasis of prostate cancer is frequent and generally manifold and osteoblastic (2). One of the major complications associated with bone involvement is severe pain, which restricts mobility and sleep, greatly reducing performance status and compromising the patient's quality of life. Conventional approaches, including external beam radiotherapy or systemic chemotherapy, are the mainstay of treatment in advanced cancers (3). However, the identification of a successful treatment for patients with huge osseous metastases is difficult. Radiopharmaceuticals are now available for the palliation of metastatic bone pain (4,5). As a pure β-emitter, strontium-89 chloride (^89^Sr) has similar biological properties to calcium, which has a natural affinity for metabolically active bone. It localizes in skeletal tissue primarily at sites of increased osteoblastic activity (6). In most cases, ^89^Sr is used for the palliation of painful osseous metastases. The nuclide also shows great potential in the therapy for osseous metastases of prostate carcinoma (7–9). The present study describes the use of ^89^Sr therapy in a patient with huge osseous metastases from prostate carcinoma where a partial response to treatment was obtained.

## Case report

**Figure 1.**
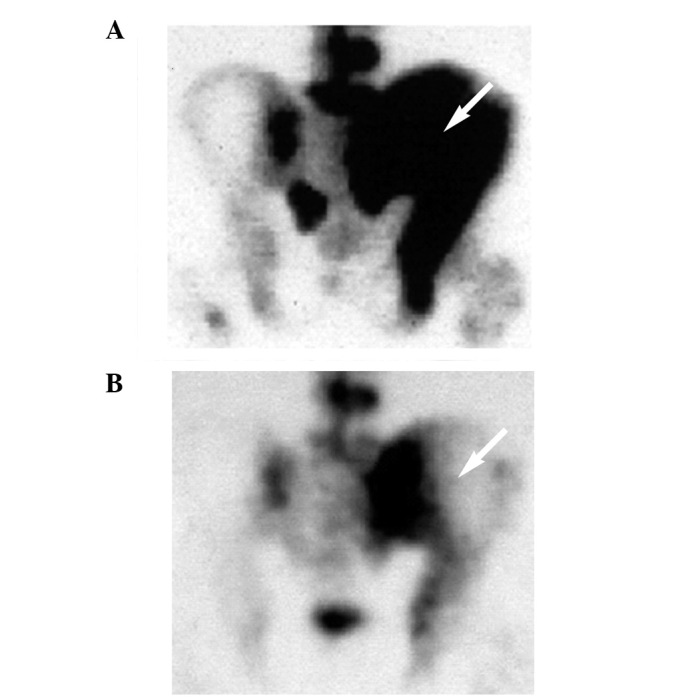
^99m^Tc-MDP bone scan obtained (A) prior to treatment showed prominent accumulation of the isotope in the right hip, ilium and ischium, and (B) post-treatment, the extent of the original lesions was markedly reduced. Arrows indicate the huge osseous metastatic lesion of the left ilium. ^99m^Tc-MDP, ^99m^Tc-methylene diphosphonate.

**Figure 2.**
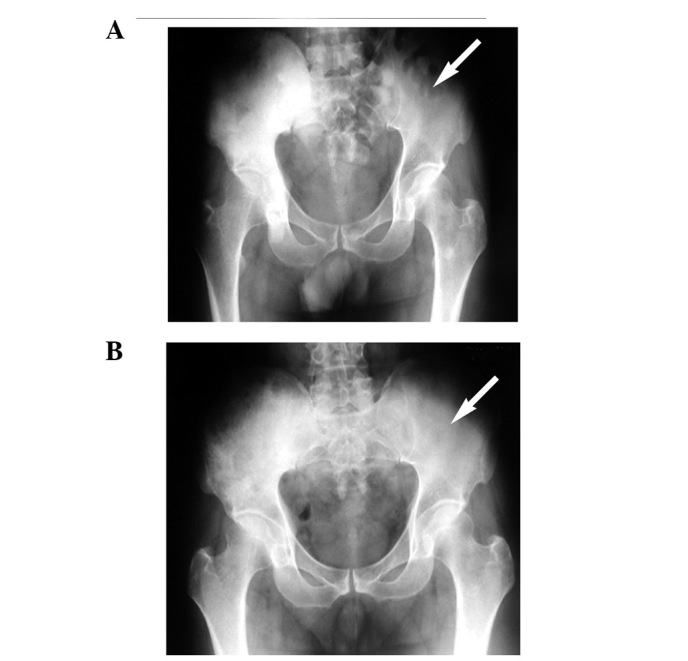
X-ray (A) prior to treatment showed marked lytic lesions corresponding with the baseline ^99m^Tc-MDP bone scan and (B) post-treatment, a new osteosclerotic response at these lytic lesions was observed. Arrows indicate the huge osseous metastatic lesion of the left ilium. ^99m^Tc-MDP, ^99m^Tcmethylene diphosphonate.

**Figure 3.**
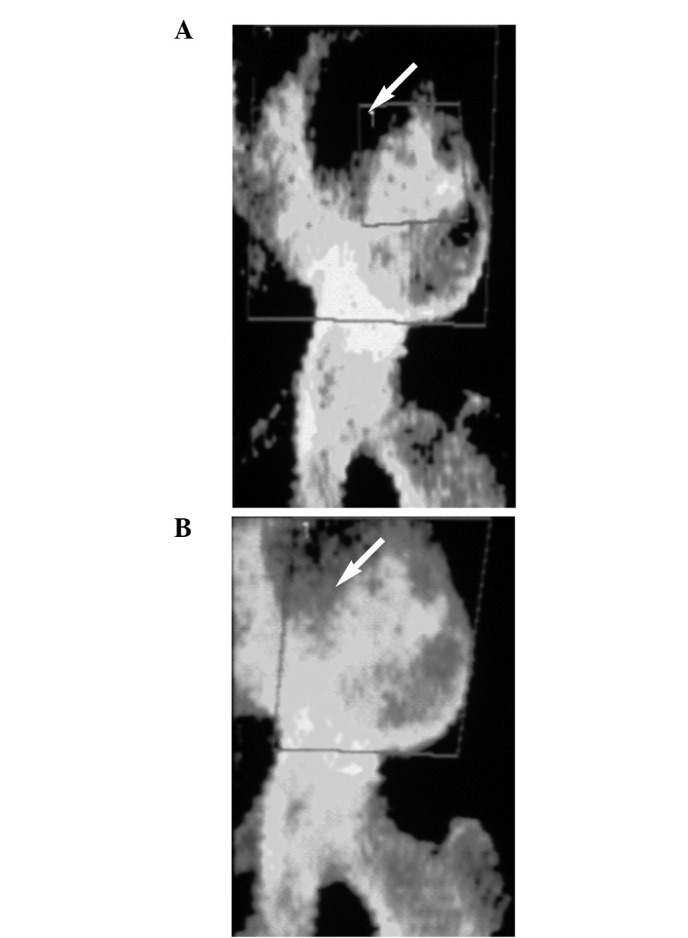
Comparison of BMD images of the left hip and ilium (A) prior to and (B) post-treatment indicated that the original lesions had been repaired and that the BMD value had increased. Arrows indicate the huge osseous metastatic lesion of the left ilium. BMD, bone mineral density.

**Figure 4.**
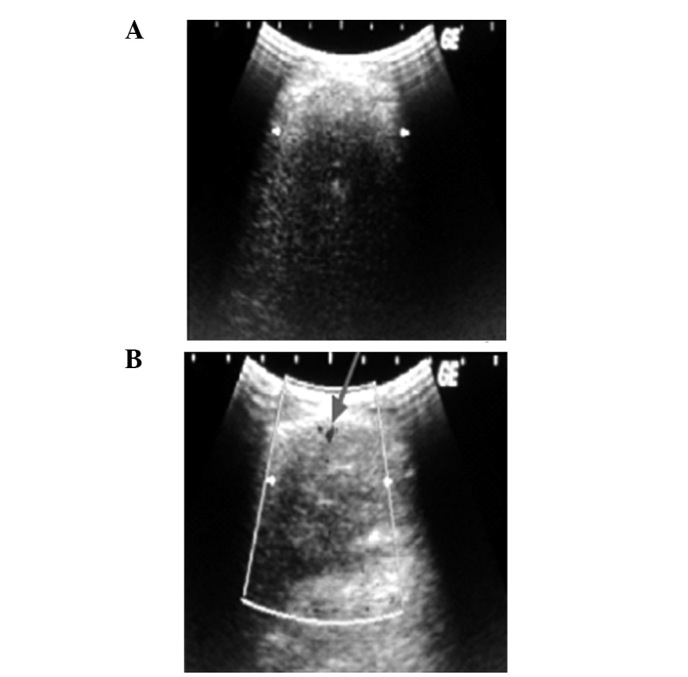
Comparison of ultrasonic images (A) prior to and (B) post-treatment showed that the original lesions had become brightly echogenic and that there was angiogenesis throughout the bone. The arrow indicates angiogenesis throughout the bone

A 52-year-old male had previously been diagnosed with prostate cancer and had been castrated (treated by orchiectomy). Following surgery, he had not been treated with external beam radiotherapy or chemotherapy. The patient presented with severe pain at multiple sites, particularly in the right hip. In the preceding six months, he had been bedridden due to a limitation of movement. A ^99m^Tc-methylene diphosphonate (^99m^Tc-MDP) bone scan showed multiple areas of abnormal uptake, including L5, S1, the sacroiliac joints, the fourth sacral vertebra and particularly the right hip, ilium and ischium which were covered with huge metastatic lesions (Fig. 1A). These corresponded with the lytic metastases that were evident on the baseline radiography (Fig. 2A). The patient's quality of life was noticeably improved following eight consecutive treatments of ^89^Sr over 3 years (each dose 4 mCi, total dose 32 mCi or 1184 MBq). An improvement in mobility was demonstrated, as evidenced by the fact that he was now able to ride a bicycle. A comparison between ^99m^Tc-MDP bone images taken prior to (Fig. 1A) and post-treatment (Fig. 1B) showed that the lesions on the left side of the fourth sacral vertebra had almost disappeared and that the range of the pelvic lesions on the right side was significantly reduced. The iliac spine was clearly visible and levels of prostate-specific antigen (PSA) had decreased by ∼69%. An X-ray revealed that the lesions were in a quiescent state. Evidence of a new osteosclerotic response at these lytic lesions was also identified (Fig. 2B). In summary, the extent of the lesions had been significantly reduced. The bone mineral density (BMD), bone mineral content (BMC) and total score (Tscore) value of the lesions prior to (Fig. 3A) and post-treatment (Fig. 3B) were as follows: BMD, 0.704 and 0.897 g/cm^2^; BMC, 13.02 and 81.78 g; and Tscore, −2.15 (62%) and −1.14 (80%), respectively. In comparison with the ultrasonic imaging results recorded prior to treatment (Fig. 4A), the results of ultrasonography post-treatment (Fig. 4B) showed that the previous lesions had become brightly echogenic and that there was angiogenesis throughout the bone. Prior to ^89^Sr therapy, the patient's white blood cell (WBC) and platelet counts were 5.8 and 109x10^9^/l, respectively. Following the last ^89^Sr therapy, his WBC and platelet counts were 5.8 and 108x10^9^/l, respectively. In this case, a reduction in leukocyte and thrombocyte levels was not observed.

The data showed that the patient had a partial response (PR) to the treatment; not only had the patient's analgesic requirements decreased by ∼75%, but his Karnofsky Performance Score (KPS) had also risen by 30%. The patient exhibited a substantial palliative response to ^89^Sr therapy. More meaningfully, the patient had survived for 4 years with few serious side-effects. The study was approved by the ethics committee of West China Hospital of Sichuan University, Chengdu, China. Written informed consent was obtained from the patient

## Discussion

Bone metastases are a severe problem in oncology, as they are usually associated with pain (10). For the majority of patients, external beam radiotherapy provides excellent palliation for localized metastatic bone pain. However, when it comes to huge osseous metastases, particularly on the spine and pelvis, its therapeutic effect is far from ideal (11,12). Chemotherapy for huge metastases has not yet been shown to confer any survival benefit, although its role in the palliation of osseous metastases is indisputable. The limitation of chemotherapy for huge metastases is that the dose that may be directed to the huge bone metastasized sites is low, and increasing the dose or using multi-drug combination therapy may induce a greater antitumor response that is less effective. The non-metastatic survival and overall survival rates are not favorable, mainly due to multi-drug resistance (13,14).

Systemic radionuclide therapy is an optimal choice for the management of intractable metastatic bone pain. ^89^Sr is a bone-seeking radiopharmaceutical used for internal radiation therapy, with characteristics summarized as follows: i) it is able to provide pure β particles (electrons) with a maximum energy of 1.49 MeV, and a mean energy of 0.58 MeV, which have an apparent tumoricidal effect; ii) the mean range of β particles in metastatic lesions is short, ∼2.4 mm, and therefore ^89^Sr is likely to cause only minor radiation damage to the bone marrow and surrounding tissues; iii) the physical half-life of ^89^Sr is 50.5 days, so it may provide long-lasting relief (6,8,9,15,16). Following intravenous administration of the usual therapeutic dose of 4 mCi (148 MBq), ^89^Sr becomes concentrated in bone in proportion to osteoblastic activity (17). The uptake of the radiation is ten times greater in metastatic tumor lesions than in healthy bone. Clinical studies have shown that irradiation with the β-rays emitted from the radionuclide *in vivo* at the site of the bone tumor, with an absorbed dose of 800 cGy per time, has an effect on pain relief that is comparable to that of high-dose, long cycle radiation therapy (6,18).

Based on the physical and metabolic properties of ^89^Sr, generally following intravenous administration, a patient's systemic reaction should be mild. In the present study no significant decrease in hematological variables associated with ^89^Sr administration was observed. Therefore, from the evaluation of the imaging results in the present study and the previously mentioned multi-disciplinary approaches, the conclusion may be made that ^89^Sr treatment of huge osseous metastases in prostate carcinoma has a curative effect and is also relatively safe.
